# Different genetic architectures underlie crop responses to the same pathogen: the {*Helianthus annuus* * *Phoma macdonaldii*} interaction case for black stem disease and premature ripening

**DOI:** 10.1186/s12870-017-1116-1

**Published:** 2017-10-19

**Authors:** Amandine Bordat, Gwenaëlle Marchand, Nicolas B. Langlade, Nicolas Pouilly, Stéphane Muños, Grégory Dechamp-Guillaume, Patrick Vincourt, Emmanuelle Bret-Mestries

**Affiliations:** 1 LIPM, Université de Toulouse, INRA, CNRS, Castanet-Tolosan, France; 2 Present address: INRA, Université de Bordeaux, UMR 1332 de Biologie du Fruit et Pathologie, CS 20032, 33882 Villenave d’Ornon, France; 3 Present address: EURALIS Semences, Domaine de Sandreau, 6 Chemin de Panedautes, 31700 Mondonville, France; 4 Terres Inovia, UMR 1248 AGIR, BP52627, F-31326 Castanet-Tolosan, France; 5 ENSAT, UMR 1248 AGIR, BP52627, F-31326 Castanet-Tolosan, France; 6 AGIR, Université de Toulouse, INRA, INPT, INP-EI PURPAN, Castanet-Tolosan, France

**Keywords:** Premature ripening, Black stem, Sunflower, *Phoma macdonaldii*, QTL mapping

## Abstract

**Background:**

*Phoma macdonaldii* has been reported as the causal agent of black stem disease (BS) and premature ripening (PR) on sunflower. PR is considered as the most widespread and detrimental disease on sunflower in France. While genetic variability and QTL mapping for partial resistance of sunflower to stem, collar and roots attacks have been reported on plantlets in controlled conditions, this work aims to describe the genetic variability in a subset of a sunflower lines, and for the first time to map QTL involved in PR resistance evaluated in field conditions using controlled inoculation.

**Results:**

An efficient and reliable method for inoculation used in field experiments induced stem base necrosis on up to 98% of all plants. A significant genetic variability for PR resistance in the field was detected among the 20 inbred lines of the core collection tested across the two years. For QTL mapping, the PR resistance evaluation was performed on two recombinant inbred lines (RIL) populations derived from the crosses XRQxPSC8 and FUxPAZ2 in two different years. QTL analyses were based on a newly developed consensus genetic map comprising 1007 non-redundant molecular markers. In each of the two RIL populations, different QTL involved in PR partial sunflower resistance were detected. The most significant QTL were detected 49 days post infection (DPI) on LG10 (LOD 7.7) and on LG7 (LOD 12.1) in the XRQxPSC8 and FUxPAZ2 RIL population, respectively. In addition, different QTL were detected on both populations for PR resistance measured between 14 and 35 DPI. In parallel, the incidence of natural attack of *P. macdonaldii* resulting in BS disease was recorded, showing that in these populations, the genetic of resistance to both diseases is not governed by the same factors.

**Conclusion:**

This work provides the first insights on the genetic architecture of sunflower PR resistance in the field. Moreover, the separate studies of symptoms on different organs and in time series allowed the identification of a succession of genetic components involved in the sunflower resistance to PR and BS diseases caused by *Phoma macdonaldii* along the development of the {plant * pathogen} interaction.

**Electronic supplementary material:**

The online version of this article (10.1186/s12870-017-1116-1) contains supplementary material, which is available to authorized users.

## Background

The sunflower crop is faced to several diseases caused by fungi and oomycetes in all the regions where it is cultivated. The use of resistant sunflower varieties is an efficient way to control the diseases, and resistance to these diseases remains a major target for sunflower breeding [1]. During the last two decades, the premature death [2] or premature ripening (PR) induced by *Phoma macdonaldii* Boerema (teleomorph: *Leptosphaeria lindquistii*) became the most severe and widespread sunflower disease in France, and could be partly responsible for the yield stagnation around 2.5 t.ha^−1^. *P. macdonaldii* is also responsible for black stem disease (BS). However, the damages of PR on seed yield appeared greater than those of black stem [3]. As the chemical control of the disease remains difficult, potentially dangerous for the environment and becomes less socially acceptable, the development of even partially resistant varieties is an important breeding objective. Some genetic variability has been described for sunflower resistance to BS disease [4, 5] and PR [3]. Several studies dealing with the genetic control of sunflower resistance to *Phoma* attacks on petiole stem base and roots of seedlings in growth chamber have demonstrated the quantitative character of this resistance [6–11]. Cytological observations of a susceptible and of a more resistant inbred line showed that the development of fungal hyphae within the stele was affected in the more resistant genotype, suggesting the involvement of a plant compound in the defense [12]. In addition, candidate genes could be identified through transcriptomic studies of the sunflower * *P. macdonaldii* interaction (e.g. sunflower-like-lipase, MYB-related transcription factor regulating PAL2, a key enzyme involved in the phenylpropanoid pathway) [13]. However, in these studies, the pathosystem was developed on two-leaf-stage plantlets that is not fully representative of natural attacks. In field or in greenhouse, *P. macdonaldii* has been found to cause higher damages when contamination occurs at the star bud phenological stage (E1) than at earlier phenological stages [14]. Recently, we demonstrated the clear role of aerial *Phoma* infection in PR compared with soilborne inoculums [15]. In addition, we showed that artificial inoculation at the stem base with pycniospores or mycelium of *P. macdonaldii* could be used for screening genotypes showing a substantial level of resistance to PR. Using this phenotyping protocol in field, we investigated in this study the phenotypical variability and the genetic architecture of premature ripening and black stem resistance along plant development in *Helianthus*.

## Methods

### Sunflower genetic material

- In 2009, the resistance level to PR of 42 genotypes was evaluated in a field trial in Auzeville-Tolosane: 40 lines were selected from the *Helianthus annuus* core collection of 48 lines [16] and two additional lines were included due to their high level of resistance in 2007 preliminary observations: (Tub-1709-1)-1-6A is derived by selfing from the USDA accession TUB-1709-1 which results from an introgression of *H. tuberosus* [17], and 97B7 is an INRA line derived from a cross involving *H. argophyllus*. According to the results, a representative subset of 21 lines was chosen to confirm their resistance level in a second trial in 2010 (Table 1).
- Two sets of RIL were used for QTL mapping: a) a subset of 117 F8 lines from the “INEDI” RIL population which was obtained by single seeds descent from a cross between the lines XRQ and PSC8 [18, 19], b) a subset of 113 F7-F10 lines from the “FUxPAZ2” RIL population, derived by single seed descent from a cross between the lines FU and PAZ2 [8, 19]. These two RIL populations were chosen because of the difference for the resistance level to premature ripening of the parental lines. Indeed, in a 2008 previous trial, the XRQ and PSC8 lines were evaluated in the field using the same protocol and the same strain of *P. macdonaldii* than those used in this work: at phenological stage M1.2-M1.3 (43 days after contamination), 27% of plants were prematurely ripened for XRQ against 77% for PSC8. In the core-collection trial in 2010, FU was one of the most susceptible lines to premature ripening (PR AUDPC = 13.73) and PAZ2 one of the most resistant (PR AUDPC = 1.61). The four parental lines confirmed therefore their differences and the corresponding RIL populations were chosen for these experiments in 2010 and 2011. If XRQ and PAZ2 appeared to be more resistant than PSC8 and FU, they don’t represent extreme behaviors towards *P. macdonaldii*. The availability of recombinant lines was also a criterion for choosing this material.

**Table 1:** Description of the 21 lines tested for their resistance to Premature Ripening. ‘Core collection ID’ indicates the line code used in this study and as previously used [16], ‘Type’ indicates if lines restore the male sterility on PET1 cytoplasm (R) or maintain the PET1 cytoplasmic sterility (B), ‘Origin’ indicates the line pedigree

Core collection ID	Type	Name	Origin	Breeder
SF056	B	FU	Romanian line x Russian line	INRA
SF060	B	G 2789	American line x Argentine line	INRA
SF061	B	GIZ	Selection from Egyptian population	INRA
SF063	B	H 101 22	Selection from Moroccan population	INRA
SF085	B	CD	HA89 selection	USDA
SF107	B	92A6	*H. argophyllus* x French line	INRA
SF110	B	NF	Genic male sterility selection from Russian population Armavir	INRA
SF193	B	XRQ	HA89 x Russian population Progress	INRA
SF263	R	A 1786	Selection from Australian breeding population	INRA
SF278	R	OQP7	*H. argophyllus* x recurrent selection x RHA345	INRA
SF292	R	PRS5	Rumanian line x Russian line x RHA271	INRA
SF302	R	PAC2	*H. petiolaris* restorer x HA61	INRA
SF306	R	PAZ2	Serbian line x French line x Zambia population	INRA
SF308	R	PAC1	*H. petiolaris* restorer x HA61	INRA
SF310	R	PST5	Recurrent selection for Sclerotinia resistance	INRA
SF326^a^	R	PSC8	Recurrent selection for Sclerotinia resistance	INRA
SF330	R	RHA801	Composite restorer line	USDA
SF334	R	U85	Selection from USDA source for Sclerotinia resistance	INRA
SF336	R	RSCOTT	Selection from Australian breeding population	INRA
Tub-1709-1-1-6A	Unknown	TUB	Derived from an USDA accession (PI 564517) including *H. tuberosus* germplasm [17]	USDA
SF064	B	97B7	*H. argophyllus* x French line	INRA

^a^SF326 (PSC8): missing phenotyping data in core collection trials in 2009 and 2010

### Experimental design

Four main field experiments were carried out to evaluate the resistance level of sunflower lines, from the three genetic designs, against PR over 3 years (2009, 2010 and 2011) at INRA, Auzeville-Tolosane, near Toulouse (Haute Garonne, South-West, France). Before sowing, N fertilization was applied on each trial (60 kg.ha^−1^). The crop was sown at the beginning of May in 2009 and at the beginning of April in 2010 and 2011. The experiments were conducted in a randomized block design, with two replications in 2009 and three replications in 2010 for core collection trials, and with two replications for XRQxPSC8 (2010) and FUxPAZ2 (2011) RIL populations trials. In these two last experiments, the parental lines of the RIL populations were included as controls. Each plot consisted of 3 rows of about 25 plants. Plant density was 7 plants.m^−2^ after thinning. According to rainfall, irrigation (30 mm) was performed before inoculation in 2011. As *Phomopsis helianthi* is regularly present in Auzeville-Tolosane, Corbel (fenpropimorph, 0.8 l ha^−1^, BASF) was applied in June to control specifically this disease in all experiments. This fungicide does not affect the development of *Phoma macdonaldii*.

### Artificial plant inoculation

A single *P. macdonaldii* monopycniospore strain (MPH2) was used in all experiments. It was isolated from natural severe stem base lesions observed in 2006 in a sunflower crop located in Montgaillard-Lauragais (Haute-Garonne, France) and selected for its severe aggressiveness on a commercial cultivar Heliasol RM (KWS AG) susceptible to *P. macdonaldii*. Mycelium conservation, inoculum production and mycelium inoculation were performed as previously described [15]. Inoculum was produced on potato dextrose agar (PDA Difco 39 g.l^−1^,150 mg of streptomycin, pH 6) and grown at 25 °C for 10 days in the dark. Mycelium inoculation was carried out at bud stage (E1 to E5) on 15 uniform plants per plot. A 6 mm diameter disk of PDA with mycelium was placed at the stem base of each plant and immediately covered with a damp cotton and an aluminum foil to prevent dehydration and left for 5 days. No natural attack at the stem base was observed before artificial inoculation.

### Disease assessment

Disease symptoms were observed weekly on the 15 plants per replication, up to physiological maturity on the four experiments.

**Fig. 1:**
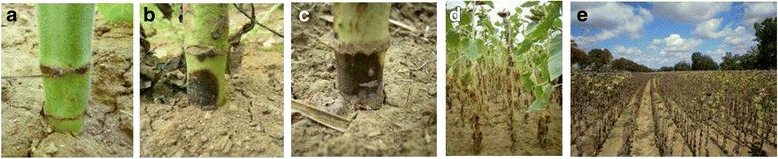
Correspondence between the scoring of *Phoma macdonaldii* PR disease and the symptoms observed on sunflower (**a**: score 0; **b**: score 1; **c**: score 2; **d**: score 3; **e**: score 4)

Development of necrosis at the stem base and premature ripening (PR) induced by the *P. macdonaldii* artificial inoculation were assessed in each experiment from 14 days post-inoculation (DPI) to 56 or 63 DPI with an assessment every week. The disease was scored using a 0–4 scale: 0 = healthy plant, 1 = less than 3/4 of the stem base circumference black, 2 = necrosis girdling the stem base, 3 = all leaves wilted but the stem green, 4 = plant completely dry (Fig. 1). A plant was defined as reaching the PR stage when it is completely dry before physiological maturity, with necrosis girdling the stem base.

As natural infection caused by *P. macdonaldii* occurred from adjacent wheat fields and induced BS symptoms on the four trials, a disease assessment was also performed on BS disease, using a 0–2 scale: 0 = healthy plant, 1 = plant with isolated spots on the stem, 2 = plants with coalescent spots, and according to the same timing than for PR assessment (once a week, at the same time than PR assessment). These symptoms are expected to be induced by primary inoculum, as the secondary cycle of de *P. macdonaldii* was never observed on the French territory, contrary to what is described by other authors [20, 21].

The developmental stage of each genotype was recorded as previously described [22] at each disease assessment.

All plants affected during the cropping season by other fungal diseases (*Phomopsis* stem canker, *Verticillium* wilt, *Alternaria* leaf spot and blight) were excluded from the PR symptoms assessment: the PR symptoms were recorded only on the remaining stem base infected plants in order to avoid any confusion between senescence due to *Alternaria* sp. or due to *P. macdonaldii*. *Alternaria* leaf spot and blight was assessed in July 2010 on XRQxPSC8 RIL population (two replications) using a 0–3 scale: 0 = healthy plant, 1 = foliar symptoms in the lower part of the plant, 2 = foliar symptoms on the whole plant, 3 = blighted plant.

In order to avoid any artifact due to the experimenter effect, a same experimenter never carried out two successive disease assessments on the same plot.

### Statistical analyses on disease assessment data and other phenotypic traits

For each experiment devoted to disease assessment and each disease assessment date or developmental stage, a mean disease score of each plot and each genotype was calculated and the genotype effect on BS and PR resistance was assessed by analysis of variance on mean disease scores according to a general linear model (GLM procedure, SAS software, SAS Institute Inc.).

The BS and PR Area Under Disease Progress Curves (AUDPC) are estimated as previously described [23]:

$$ \mathrm{AUDPC}=\sum_{i=1}^{n-1}\left(\frac{y_i+{y}_{i+1}}{2}\right)\left({t}_{i+1}-{t}_i\right) $$

where *y*_*i*_ = mean BS disease score or percentage of premature ripened plants at the *i* ^*th*^ observation, *t* = time (days) after inoculation at the *i* ^*th*^ observation, and *n* = total number of observations.

Broad-sense heritability was estimated according to the following formula: *h* ^2^ = σ^2^_*G*_/[σ^2^_*G*_ + (σ^2^_***E***_/*r*)], where σ^2^_*G*_ is the genetic variance (MS*g* – MS*gr*)/*rn*, and σ*e* ^2^ is the environmental variance (MS*e*), *n* is the number of plants, and *r* is the number of replicates.

### QTL analysis

Using the previous genotypic data and the methodology previously described [19], the two RIL populations were genotyped with a complementary AXIOM array (Affymetrix, USA) composed of 197,863 SNPs. For the “INEDI” population, we used a set of 32,666 SNPs that were polymorphic (with no segregating distortion in the whole population) and showed the highest quality (“PolyHighResolution”) according to the automatic allele calling from Axiom Analysis Suite software. In the same manner, a set of 28,529 SNPs from the FUxPAZ2 RILpopulation was used for mapping. A set of SSR markers and additional SNPs from candidate genes sequencing was added to the “AXIOM” markers. A consensus map was then built with the CARTHAGENE software (http://www.inra.fr/mia/T/CartaGene/, [24]) in using *mergen* command. In the process of obtaining robust consensus map, we used a) the markers mapped in [19], b) and from the AXIOM array, only the markers being genotyped in both populations. During the map building, only the markers with a LOD 2 points greater than 10 were selected.

For each RIL population, two different maps derived from this consensus map were used to perform QTL detection with MCQTL [25]. The phenotypic traits were recorded in the following independent trials: disease traits with artificial (PR) or natural (BS, *Alternaria*) infection for the “INEDI” RIL population (2010), diseases traits with artificial (PR) or natural (BS) infection for the FUxPAZ2 RIL population (2011).

A threshold corresponding to a Type I error rate of 1% at the genomewide level was used, as determined after 3000 replications of the resampling process for each trait and averaging the limit value for QTL detection across the different traits. The supports of the QTL were determined using the software MCQTL [25] with the QTL LOD value minus 2, which is expected to provide a 95% confidence. This procedure is producing longer interval supports that often reported in the literature (for example [9, 10]).

## Results and discussion

### Disease development in the four experiments

BS and PR development data were obtained on 1086 plants for 20 lines of the core collection in 2010, 2919 plants in the RIL population XRQxPSC8 and 3153 plants in the RIL population FUxPAZ2.

#### Stem base necrosis and PR

In the four experiments, the mean percentage of plants showing *Phoma* necrosis at the stem base (disease score ≥ 1) at 14 DPI ranged from 97.9% to 98.7%. These high percentages showed the effectiveness of the inoculation method. Similar results were previously observed in field and greenhouse trials, both on adult plants [3, 15].

In all four experiments, stem base necrosis increased from disease score 1 to disease score 2 during the first 35 DPI. All genotypes presented at least one plant with a girdling *Phoma* necrosis at the stem base (disease score = 2). The first premature ripened plants (disease score = 4) appeared in each trial at 42 DPI and between 17% and 33% of plants showed PR at 49 DPI according to the experiments. One week later, this percentage ranged between 35% to 51%. This evolution is consistent with previous results obtained on two commercial sunflower hybrids in field trials [3] and greenhouse trials [15], where the premature ripened plants started to appear at 43 DPI.

#### Black stem disease

Natural attacks of black stem disease occurred in the four trials. At the beginning of the assessment of disease resistance (14 days post inoculation at the stem base), the average percentage of plants with BS symptoms ranged from 0% (core-collection trial in 2009) to 74% (FUxPAZ2 trial in 2011) over the 3 years. The black stem disease progressed along plant development and the mean disease score reached 1.57 in core-collection trial in 2010, 1.50 in XRQxPSC8 RIL population experiment in 2010 and 1.93 in FUxPAZ2 RIL population experiment in 2011 respectively at the last observation (Table 2). A high variability between genotypes was observed in each experiment (Table 2, Additional file 1).

**Table 2:** Statistical results of *Phoma macdonaldii* black stem disease and premature ripening scores for the two sunflower RILs populations XRQxPSC8 and FUxPAZ2 and their parental lines

Variable (unit)	Mean of the parental lines		RILs + parental lines			
	XRQ	PSC8	Mean	SD	Range	CV (%)
Pm_BS_dpi14 (score unit)	0.16	0.50	0.22	0.27	0–1.33	126.0
Pm_BS_dpi21 (score unit)	0.31	1.00	0.53	0.33	0–1.67	61.9
Pm_BS_dpi28 (score unit)	0.63	1.00	0.69	0.35	0–2.00	51.2
Pm_BS_dpi35 (score unit)	0.78	1.00	0.82	0.36	0–2.00	44.0
Pm_BS_dpi42 (score unit)	1.02	1.00	1.03	0.39	0–2.00	37.9
Pm_BS_dpi49 (score unit)	1.20	1.25	1.25	0.43	0.10–2.00	34.8
Pm_BS_dpi56 (score unit)	1.32	1.25	1.40	0.40	0.14–2.00	28.7
Pm_BS_dpi63 (score unit)	1.51	1.25	1.50	0.39	0.14–2.00	25.8
AUDPC_BS (score unit.day)	46.11	67.38	48.84	15.95	6.0–86.33	32.6
Pm_PR_dpi14 (score unit)	1.22	2.00	1.64	0.28	0.86–2.00	16.9
Pm_PR_dpi21 (score unit)	1.69	2.00	1.81	0.20	1.00–2.00	11.1
Pm_PR_dpi28 (score unit)	1.75	2.00	1.90	0.15	1.21–2.07	8.1
Pm_PR_dpi35 (score unit)	1.90	2.00	1.98	0.19	1.45–3.17	9.4
Pm_PR_dpi42 (score unit)	2.00	3.50	2.19	0.43	1.50–3.93	19.6
Pm_PR_dpi49 (score unit)	2.28	4.00	2.55	0.66	1.60–4.00	25.8
Pm_PR_dpi56 (score unit)	2.79	4.00	3.03	0.71	1.67–4.00	23.4
Pm_PR_dpi63 (score unit)	3.42	4.00	3.47	0.61	1.90–4.00	17.5
AUDPC_PR (% PR plants.day)	3.67	21.7	6.87	6.13	0–23.92	89.3
	FU	PAZ2	Mean	SD	Range	CV (%)
Pm_BS_dpi14 (score unit)	0.95	0.83	0.74	0.34	0–1.21	45.3
Pm_BS_dpi21 (score unit)	1.00	1.00	1.00	0.09	0.60–1.57	8.6
Pm_BS_dpi28 (score unit)	1.03	1.03	1.07	0.17	0.86–1.87	15.4
Pm_BS_dpi35 (score unit)	1.36	1.20	1.30	0.32	0.90–2.00	24.6
Pm_BS_dpi42 (score unit)	1.97	1.73	1.63	0.36	1.00–2.00	22.3
Pm_BS_dpi49 (score unit)	2.00	1.97	1.87	0.25	1.00–2.00	13.1
Pm_BS_dpi56 (score unit)	2.00	2.00	1.93	0.17	1.00–2.00	9.0
AUDPC_BS (score unit.day)	61.74	58.33	57.47	7.40	38.50–75.25	12.9
Pm_PR_dpi14 (score unit)	1.34	1.77	1.39	0.35	0.43–2.00	25.4
Pm_PR_dpi21 (score unit)	1.76	1.95	1.64	0.32	0.71–2.07	19.8
Pm_PR_dpi28 (score unit)	1.97	1.97	1.81	0.27	1.00–2.42	15.1
Pm_PR_dpi35 (score unit)	2.26	1.99	1.92	0.27	1.00–3.00	13.8
Pm_PR_dpi42 (score unit)	3.16	2.03	2.29	0.55	1.00–4.00	23.9
Pm_PR_dpi49 (score unit)	3.71	2.13	2.90	0.69	1.14–4.00	23.9
Pm_PR_dpi56 (score unit)	4.00	2.48	3.37	0.57	1.57–4.00	17.1
AUDPC_PR (% PR plants.day)	10.99	0.57	4.23	4.43	0–17.50	104.75

*SD* standard deviation, *CV* coefficient of variation (%)

### Phenotypic variability in the core-collection

As the total number of plants per inbred line was low in 2009 (mean plant number = 13.6) compared to 2010 (mean plant number = 54.3), we chose to comment mainly on the results of the second year of evaluation.

#### Stem base necrosis and premature ripening in the core-collection

**Fig. 2:**
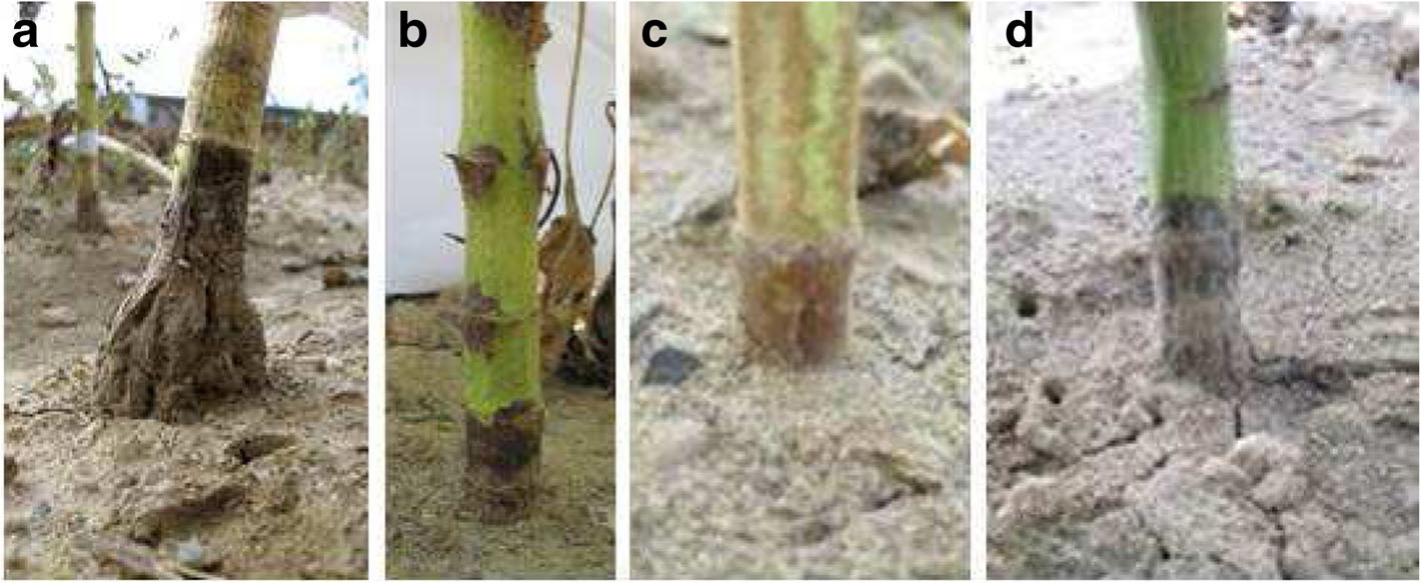
Phenotypic variability of the symptoms of *Phoma macdonaldii* at the stem base observed on the sunflower core-collection trial in 2010 (**a**: SF061; **b**: SF057; **c**: SF334; **d**: SF110)

The stem base necrosis due to *Phoma macdonaldii* infection showed a very high phenotypic variability among the sunflower lines (Fig. 2).

**Fig. 3:**
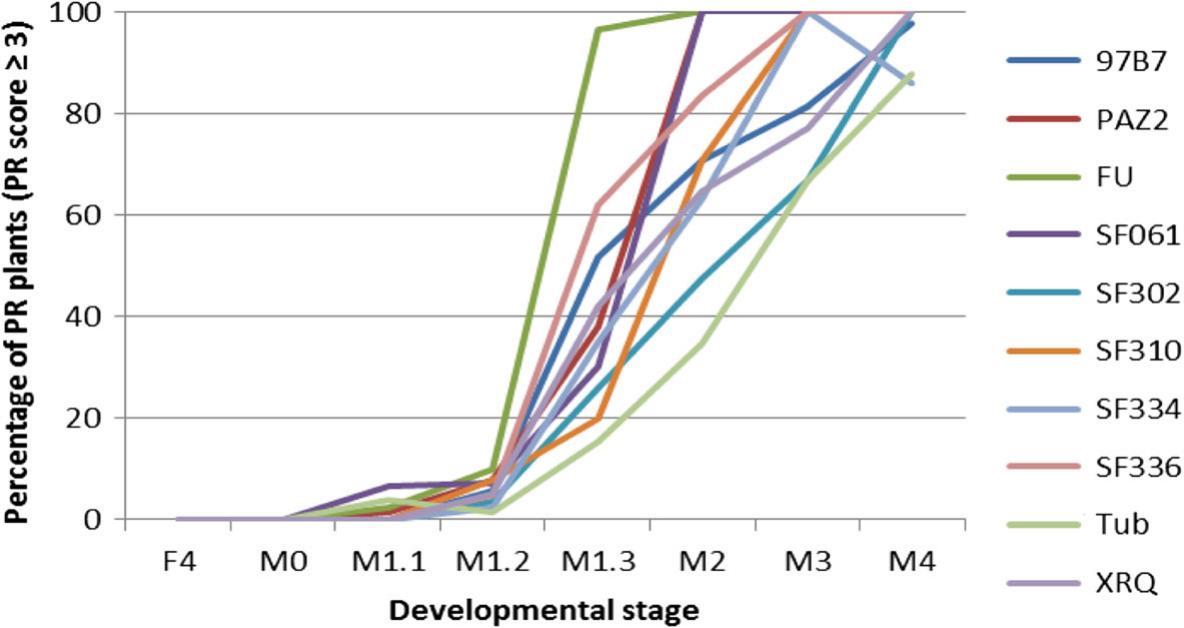
Evolution of the *Phoma macdonaldii* PR plants percentage (disease score ≥ 3) for a subset of the 20 sunflower inbred lines of the core-collection trial (2010) between the end of flowering (F4) and the complete maturity (M4) developmental stages

In the 20 lines present in the two core-collection trials, the percentage of PR plants (score = 4) at 63 DPI was on average 68% in 2009 and 55% in 2010, with a range from 0 to 100% each year. However, due to large developmental differences among the 20 lines, PR must be considered at the same developmental stage for each genotype: the M1.2-M1.3 stages allow a good evaluation of PR resistance, avoiding confusion of premature ripening with natural senescence. According to the genotypes, these stages are reached in 2010 between 35 and 63 DPI (Fig. 3).

The PR percentage at the M1.3 stage (disease score ≥ 3) ranged from 14% to 97% in 2010 (Fig. 3), with a highly significant genotype effect (*p*-value < 2 × 10^−16^).

**Fig. 4:**
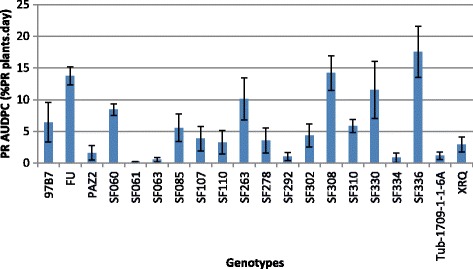
Variability of AUDPC for premature ripening observed on the 20 core-collection lines screened in 2010. The size of the errors bars equals to one standard deviation

In 2010, the PR AUDPC ranged from 0.23% PR plants.day to 17.53% PR plants.day (mean = 5.88% PR plants.day; Fig. 4), with a significant genotype effect (*p*-value = 0.00404). The more susceptible genotype seemed to be SF336 (17.53% PR plants.day) and the more resistant one SF061 (0.23% PR plants.day). The parental lines PAZ2 and XRQ are characterized by a small PR AUDPC (respectively 1.61 et 2.94% PR plants.day), as well as Tub-1709-1-1-6A, already seen resistant to the disease in a preliminary experiment (1.14% PR plants.day); in contrast, the parental line FU confirmed its susceptibility to premature ripening (13.73% PR plants.day).

A correlation analysis (Kendall rank concordance test) between core-collection lines datas 2009 and 2010 on the mean genotype values of the premature ripening disease score at each phenological stage (in order to compare the genotypes in the same conditions) was performed. Of 13 of the 14 lines for which we observed at least 10 plants in 2009, the Tau b coefficient of Kendall is greater than 0.75 and is significant at the 1% threshold. Three of the four parental lines of the RIL populations developed for QTL analysis belong to this set of lines: FU, PAZ2 and XRQ (Table 3).

**Table 3:** Results of the Kendall rank concordance tests for 14 sunflower inbred lines of the core-collection, between their premature ripening disease score assessed in 2009 and in 2010

Genotype	Data number	Tau b Kendall coefficient	*P*-value
FU	8	0.92857	0.0013
PAZ2	12	0.77865	0.0005
SF085	11	0.85985	0.0003
SF107	12	0.80918	0.0003
SF110	13	0.96776	<0.0001
SF263	12	0.86638	0.0001
SF278	12	0.85948	0.0001
SF292	12	0.55705	0.0194
SF302	12	0.81989	0.0007
SF308	11	0.82776	0.0013
SF330	9	0.89496	0.0014
SF334	9	0.8889	0.0008
SF336	7	1.0000	0.0016
XRQ	11	0.94388	<0.0001

#### Black stem disease in the core-collection

Previous studies have shown the relevance of observing natural attacks for experiments related to the study of BS resistance variability of commercial hybrids [5] or to the evaluation of yied losses [26], with very consistent results between 2 years of experiments.

**Fig. 5:**
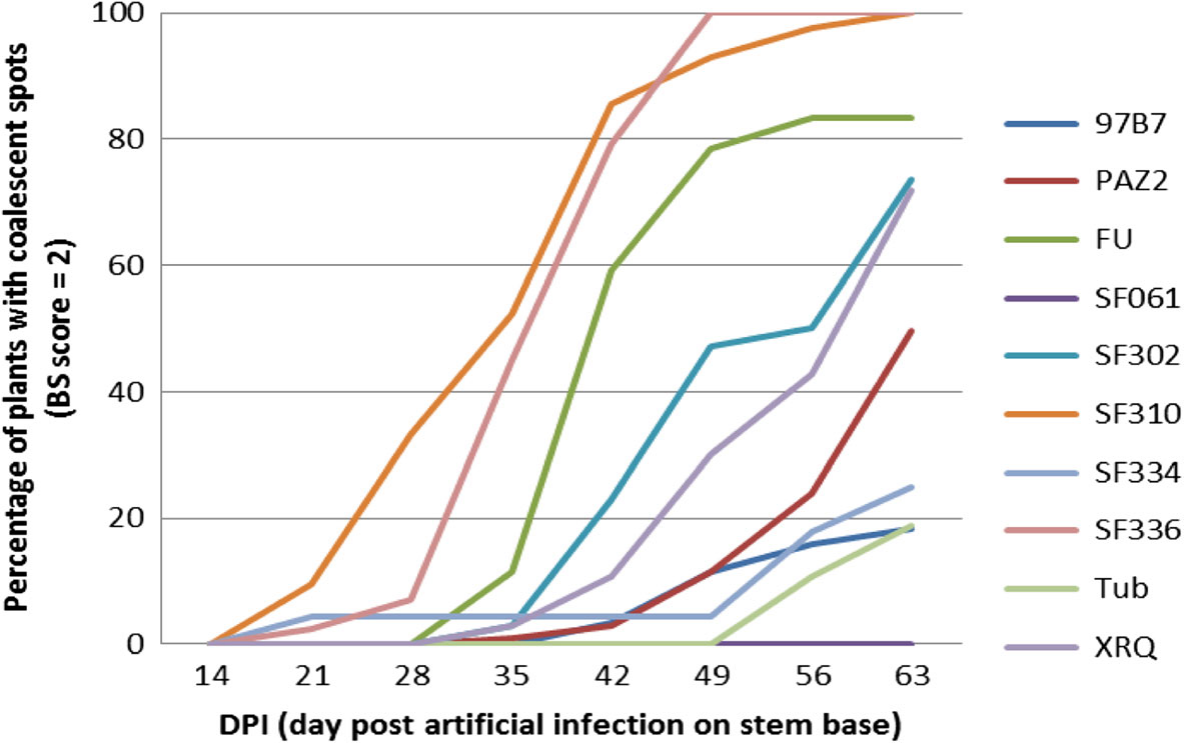
Evolution of the *Phoma macdonaldii* BS disease (score 2 = percentage of plants with coalescent spots on the stem) for a subset of the 20 sunflower inbred lines of the core-collection trial (2010)

In our experiment of 2010, at 14 DPI, the percentage of plants without any symptom on the stem under natural attack ranged from 14% for the line SF310 to 100% for SF061 (mean = 74%). The evolution of the percentage of plants with coalescent spots (Fig. 5) showed high differences in behavior between lines: SF310 and SF336 exhibited a very fast evolution of this symptom, with more than 80% of plants affected at 42 DPI. In contrast, lines Tub, 97B7 and SF334 had less than 30% of their plants with coalescent spots at 63 DPI. The SF061 line seemed to be very resistant to BS disease with 93% of healthy plants at the end of the experiment.

At each disease assessment, the analysis of variance of the mean BS disease score showed a highly significant genotype effect (*p*-value from 2.77*10^−9^ to 0.00211), showing that the disease scoring was efficient to reveal genetic variability. This confirms the previous results obtained in field trials on six commercial hybrids [5] and on 54 other inbred lines [4].

### Disease observations on RILs

#### Stem base necrosis and premature ripening on RILs

**Fig. 6:**
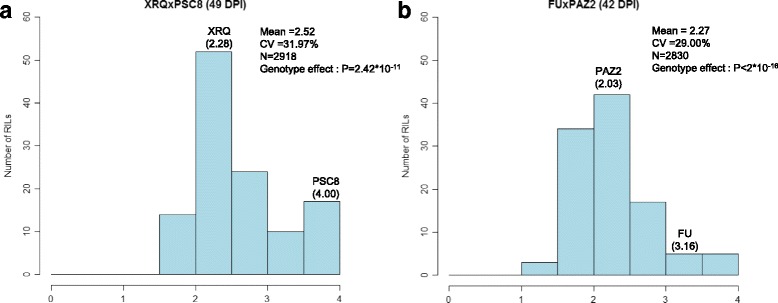
Frequency distributions of mean disease score for PR resistance in the XRQxPSC8 (**a**) at 49DPI and FUxPAZ2 (**b**) at 42 DPI RIL populations. Phenotypic values of parental lines are indicated

In the two RIL populations, the PR disease score increased in the same manner from 14 dpi to 56 or 63 dpi (Table 2). The mean of AUDPC for PR confirmed the difference between the parental lines (XRQ and PAZ2 more resistant than PSC8 and FU). In the two RIL populations, the developmental stage M1.2-M1.3 was reached between 42 and 49 DPI. At this developmental stage, the global mean disease score of XRQxPSC8 and FUxPAZ2 RIL populations was respectively 2.52 (CV = 32%) and 2.27 (CV = 29%) (Fig. 6). Within the two RIL populations, this mean disease score varied from 1.60 to 4.00 for XRQxPSC8 and 1.00 to 4.00 for FUxPAZ2. These data confirmed the behavior of the four parental lines: PSC8 and FU appeared quite susceptible (4.00 and 3.16 respectively), whereas disease severity remained low on XRQ and PAZ2 (2.28 and 2.03 respectively).

The mean disease score analysis of variance showed significant to highly significant differences between inbred lines in the XRQxPSC8 population (*p*-value from 0.0111 to 6.42*10^−13^) and highly statistically significant differences between inbred lines in the FUxPAZ2 population (*p*-value from 1.54*10^−11^ to *P* < 2*10^−16^) according to the disease assessment date. In the two RIL populations, highly significant differences were also highlighted for AUDPC_PR (*P* < 0.0001).

More details are available in Additional file 1.

In the two RIL populations, broad-sense heritability (Table 4) appeared moderate to large: respectively from 0.36 to 0.76 in the XRQxPSC8 population, from 0.73 to 0.86 in the FUxPAZ2 population. Although they were probably overestimated because the measurements were made in only one environment, these values show a certain reliability in the measured character which is a very important result considering the time required for the expression of the symptoms on adult plants.

**Table 4:** Broad-sense heritabilities of the resistance to *Phoma macdonaldii* black stem disease and premature ripening in the two sunflower RIL populations XRQxPSC8 and FUxPAZ2

	XRQxPSC8	FUxPAZ2
Black stem disease		
Pm BS_dpi14	0.29	0.39
Pm_BS_dpi21	0.57	0.04
Pm_BS_dpi28	0.65	0.32
Pm_BS_dpi35	0.65	0.75
Pm_BS_dpi42	0.64	0.85
Pm_BS_dpi49	0.64	0.88
Pm_BS_dpi56	0.57	0.75
Pm_BS_dpi63	0.49	–
AUDPC_BS	0.70	0.88
Premature ripening		
Pm_PR_dpi14	0.44	0.73
Pm_PR_dpi21	0.37	0.76
Pm_PR_dpi28	0.36	0.81
Pm_PR_dpi35	0.58	0.74
Pm_PR_dpi42	0.76	0.82
Pm_PR_dpi49	0.74	0.84
Pm_PR_dpi56	0.65	0.86
Pm_PR_dpi63	0.60	–
AUDPC_PR	0.79	0.84

#### Black stem disease on RILs

At the latest date of PR assessment, the global mean disease score of XRQxPSC8 and FUxPAZ2 RIL populations reached respectively 1.50 (CV = 26%) and 1.93 (CV = 9%). It increased 1.2 point between the first disease assessment (14 DPI) and the last notation for both RIL populations (Table 2). The parental lines seemed to be different at the same time on their mean disease score at 14 DPI and on the evolution of the severity of the disease (AUPDC). At each assessment date, significant to highly significant differences between inbred lines have been highlighted in the two RIL populations except at 21 DPI in FUxPAZ2 population (Additional file 1).

In the FUxPAZ2 population, broad-sense heritability for black stem disease in our field trial ranged from 0.04 to 0.88 according to the disease assessment date and reached 0.88 for AUDPC_BS (Table 4). When the black stem disease is well established (from 42 DPI, mean value of BS disease score = 1.02), the estimated heritability values range between 0.75 and 0.88 and are close to that previously obtained (0.94) in F2-F3 families of the same population under controlled conditions on plantlets [8]. In the XRQxPSC8 population, h^2^ ranged from 0.29 to 0.65 and reached 0.70 for AUDPC_BS. While these estimates are probably over-valued because we have only one experiment, these results indicate that a large part of the variation of black stem disease progression was due to genetic factors.

### QTL mapping for the resistance to premature ripening and black stem disease

One thousand seven markers (genetic bins) were mapped on the consensus map: 155 SSR or INDEL, 8 markers derived from Resistance Gene Candidates Genes (RGC), 231 SNP on Candidate Genes other than RGC [19], 599 SNP from anonymous AXIOM sequences, 9 markers derived from BAC End sequences, 5 phenotypic markers. Primers for CG and RGC have been previously made available (https://www.heliagene.org/Web/public/mapping_downy_mildew_resistance_genes.html). Marker information on SSR are publicly available. Context sequences for AXIOM markers are made available in Additional file 2. Five hundred seventy nine markers were mapped on the FUxPAZ2 RIL population, while 934 markers were mapped on the “INEDI” RIL population (Additional file 3).

**Fig. 7:**
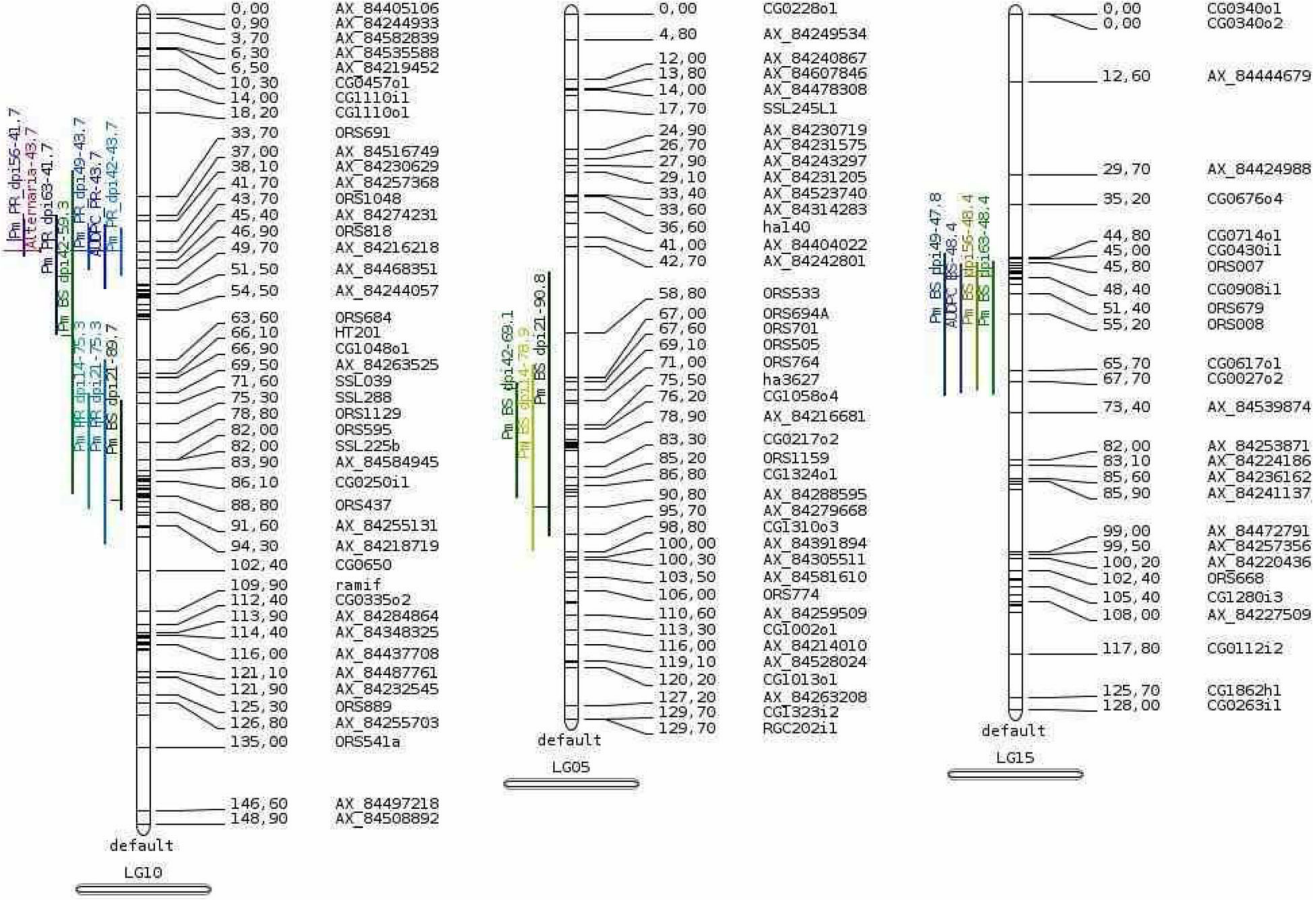
Mapping of QTL detected for all the traits in the “INEDI” RIL population. Premature Ripening Traits (Pm_PR_dpiN for *Phoma macdonaldii* PR score N days post inoculation, AUDPC_PR), Black Stem Disease Traits (Pm_BS_dpiN for *P.macdonaldii* BS score N days post inoculation), Alternaria for susceptibility score to *Alternaria helianthi*

In the “INEDI” population, different QTL were mapped depending on the type of disease (PR or BS). For the PR related traits, two different QTL were identified for early symptoms (Table 5, Fig. 7): a first QTL (Pm_PR_dpi14) was mapped on LG16 for early symptoms (14 DPI) accounting for 28.8% of the phenotypic variability, and another one was mapped on LG10 (Pm_PR_dpi14 & 21) accounting for 19.1 to 21% of the phenotypic variability. For the latest symptoms (42 to 63 dpi), one QTL (Pm_PR_dpi42 to 63, AUDPC_PR) is mapped on LG10, accounting from 16 to 26.9% of the phenotypic variability. For the BS related traits, QTL were mapped on LG5 (Pm_BS_dpi14 & 21) and LG10 (Pm_BS_dpi21) for early symptoms. For later symptoms (42 to 63 DPI), three QTL (Pm_BS_dpi 42 to 63, AUDPC_BS) are detected on LG5, LG10 and LG15. Each of these last QTL accounted between 15.6% and 19.9% of the phenotypic variability. The line XRQ was found carrying the resistance allele for PR related traits, and generally the susceptibility allele for the BS related traits.

**Table 5:** QTL detected (Type I error rate of 1% at the genomewide level) for Black Stem disease (BS) and Premature Ripening (PR) disease resistance in XRQxPSC8 and FUxPAZ2 RIL populations

Disease	RIL	Trait	LG	position	min	max	R2	MCQTL test	LOD	P1-P2 contrast:value	unit
BS	XRQ-PSC8	Pm_BS_dpi14	5	78.8	64.8	99.1	18.3	4.99	4.71	0.10	score unit
		Pm_BS_dpi21	5	90.8	48.5	97.1	18.8	5.01	4.74	0.12	score unit
		Pm_BS_dpi21	10	89.7	66.1	98.2	15.7	4.19	3.80	−0.10	score unit
		Pm_BS_dpi42	5	69.1	67.7	89.5	19.7	5.29	5.07	0.14	score unit
		Pm_BS_dpi42	10	59.3	28.8	88.5	15.6	4.21	3.82	−0.14	score unit
		Pm_BS_dpi49	15	47.8	44.0	70.5	16.9	4.58	4.23	0.14	score unit
		Pm_BS_dpi56	15	48.4	45.6	69.3	19.9	5.37	5.16	0.15	score unit
		Pm_BS_dpi63	15	48.4	45.4	70.1	19.3	5.22	4.98	0.14	score unit
		AUDPC_BS	15	48.4	46.1	70.0	19.6	4.83	4.52	6,10	score unit.day
	FU-PAZ2	Pm_BS_dpi28	6	37.7	26.4	52.4	16.8	4.4	4.01	−0.06	score unit
		AUDPC_BS	6	37.7	25.5	54.2	17.7	4.3	3.93	−3,70	score unit.day
PR	XRQ-PSC8	Pm_PR_dpi14	10	75.3	69.9	91.4	21.0	5.65	5.51	−0.09	score unit
		Pm_PR_dpi14	16	79.4	76.9	94.9	28.8	7.85	8.36	0.10	score unit
		Pm_PR_dpi21	10	75.3	65.2	91.5	19.1	5.13	4.88	−0.06	score unit
		Pm_PR_dpi42	10	43.7	39.6	48.4	22.2	6.00	5.94	−0.22	score unit
		Pm_PR_dpi49	10	43.7	39.3	47.4	26.9	7.35	7.68	−0.36	score unit
		Pm_PR_dpi56	10	41.7	37.9	45.0	24.0	6.51	6.58	−0.32	score unit
		Pm_PR_dpi63	10	41.7	37.0	59.1	16.0	4.37	4.00	−0.21	score unit
		AUDPC_PR	10	43.7	38.9	51.1	23.9	6.48	6.53	−3.32	% PR plants.day
	FU-PAZ2	Pm_PR_dpi21	13	112.9	102.4	113.0	17.1	4.5	4.12	−0.13	score unit
		Pm_PR_dpi28	13	112.9	0.0	113.0	16.8	4.4	4.02	−0.15	score unit
		Pm_PR_dpi35	13	103.6	100.8	113.0	24.8	6.2	6.22	−0.13	score unit
		Pm_PR_dpi42	7	42.5	36.7	46.7	25.0	6.2	6.22	0.27	score unit
		Pm_PR_dpi49	7	42.5	38.0	46.0	39.4	10.2	12.12	0.42	score unit
		Pm_PR_dpi56	7	42.5	38.0	46.4	33.0	8.3	9.20	0.33	score unit
		AUDPC_PR	7	41.9	37.1	46.3	32.4	8.0	8.72	2.60	% PR plants.day
Alternaria	XRQ-PSC8	Alternaria	10	43.7	42.9	44.0	52.8	16.29	22.85	−0.70	score unit

Pm: *Phoma macdonaldii*; P1-P2: XRQ-PSC8 or FU-PAZ2

In the trial of 2010, the “INEDI” RIL population was affected by *Alternaria* leaf spot and blight, with very highly statistically significant variation between RIL (Kruskal-Wallis test: *P* < 0.0001). A QTL related to susceptibility to *Alternaria* and explaining 52.8% of the phenotypic variation was found strictly colocalizing with the QTL of PR late symptoms traits (Pm_PR_dpi42, 49, 56 & 63) on LG 10. The parental line XRQ was also carrying the resistance allele to *Alternaria* and for the four QTL associated to PR resistance (Pm_PR_dpi42, 49, 56 & 63; AUDPC_PR) on this LG. One might suspect an artefact due to a mis-scoring of PR resistance; however, the plants showing *Alternaria* spots were discarded from the analysis of PR resistance, and the symptoms of the stem base necrosis due to *Phoma macdonaldii* and of the leaf spots due to *Alternaria* are clearly distinguishable. Therefore, we cannot exclude a co-location of the QTL involved in both diseases or even the involvement of a common genetic factor in the resistance to both fungi. Three QTL associated to resistance to *Alternaria* blight have been recently identified under natural infection in Argentina, and their effect has been confirmed in two genetic backgrounds. One of these QTL was located on LG 10 [27], consolidating our result.

**Fig. 8:**
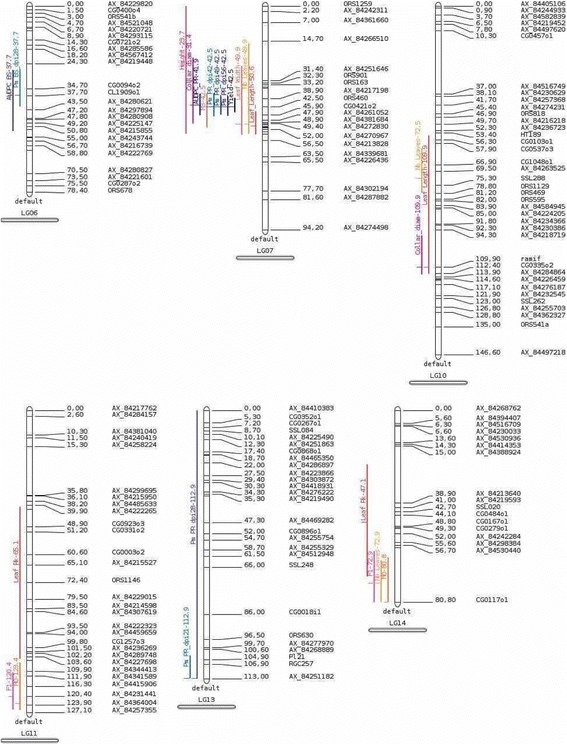
Mapping of QTL detected for all the traits in the FU x PAZ2 RIL population. Premature Ripening Traits (Pm_PR_dpiN for *Phoma macdonaldii* PR score N days post inoculation, AUDPC_PR), Black Stem Disease Traits (Pm_BS_dpiN for *P.macdonaldii* BS score N days post inoculation, AUDPC_BS) and, according to Additional file 4: Developmental traits (F1, M0, M3), morphological traits (Nb_leaves, Leaf_Rk for the rank of the biggest leaf, Leaf_Length, Leaf_width, Height, Collar_diam for base stem diameter), Yield *per se*

In the FUxPAZ2 population (Table 5, Fig. 8), QTL associated to PR were detected on LG13 for disease score at 21, 28 and 35 DPI (Pm_PR_dpi 21, 28&35) accounting for 17.1 to 24.8% of the phenotypic variability (LOD from 4.02 to 6.22), and on LG7 for disease score at 42, 49 and 56 DPI (Pm_PR_dpi 42, 49 & 56) as well as for AUDPC_PR, accounting for 25.0 to 39.4% of the phenotypic variability (LOD from 6.22 to 12.12). The line FU (susceptible to PR) was found carrying the allele associated to susceptibility for PR for the QTL associated to disease progression during grain filling (from 42 dpi) (Pm_PR_dpi 42, 49&56) while this allele is carried by the other parental line PAZ2 in the early stages of the disease (Pm_PR_dpi21, 28 & 35). These last QTL detected on LG13 during the early stage of disease development was found located in the same area as the well-known cluster of Resistance Gene Candidate [28] where race specific resistances to the Downy Mildew caused by *Plasmopara halstedii* have been mapped, and more precisely close to *Pl21* [19]. In contrast, the QTL detected during the late stages of disease development (Pm_PR_dpi 42, 49 & 56) were found located on LG7.

Four QTL involved in *Phoma* resistance to stem attack on 19-day-old seedlings on FUxPAZ2 F2-F3 families have been previously detected [8]. These QTL were mapped on linkage groups 3, 8, 11 and 12 (16, 1, 14 and 15 in the denomination they used according to [29], respectively). Several QTL have been mapped for resistance to stem and root infection in a RIL population derived from the cross RHA266*PAC2, after contaminating the plantlets with respectively four [6] or three [9] different *P. macdonaldii* isolates. Both isolate-specific and isolate-non-specific QTL were identified. The QTL involved in the isolate-non-specific resistance to stem base inoculation was mapped on LG6 [6] and LG5 and LG15 [9] while other QTL with poor specificity were located on LG5, LG13 and LG15 [6] and LG5 [9]. These four linkage groups have been found carrying QTL of resistance on adult plants in our study. In a F2-F3population from a cross between two other sunflower lines, one isolate-non-specific QTL for resistance to petiole inoculation of plantlets was also mapped on LG5 [10]. Unfortunately, there are not enough common markers between these five maps to allow a more precise comparison. In our study, no major QTL was mapped which is consistent with the quantitative nature of the resistance. No common QTL was observed between the two RIL populations. However, there is a common trend: a) in each population, QTL involved in BS and in PR diseases are not the same, b) not the same QTL are involved in early and later stage of disease development. The existence of specific QTL associated to *Sclerotinia sclerotiorum* sunflower resistance of different organs at the adult developmental stage has also been demonstrated [8, 30].

The QTL mapping work has been done from 1 year data. However, in further studies on the H. annuus * P. macdonaldii interaction [31], eight hybrids made in crossing the susceptible parental line FU and RILs carrying either resistant or susceptible alleles at the mapped QTL (LG7 and LG13) involved in PR were evaluated in 2013 and 2014 under natural infection for the development of phoma necrosis at the stem base. These hybrids, as well as their parental lines, appeared to be well classified in terms of resistance or susceptibility. This result represents at least indirect proof that some confidence could be given to our QTL.

### Relationship between morphological, developmental traits and resistance traits

In different crops, it has been found that environmental or crop management variation on one side (for example [32]), or genotypic variation on the other side (for example [33]) might result in a modification of morphological and developmental traits which affects the crop susceptibility to diseases. As such traits were recorded on the FU*PAZ2 RIL population in the frame of a supplementary experiment (2011, see Additional file 4), we compared the genetic architectures of morphological and developmental traits – including seed yield –, and disease (PR, BS) related traits. A QTL accounting for 26.7% of the phenotypic variation for the grain yield (in open pollination, *per se* evaluation) was found co-localized with the QTL of PR resistance on LG7, with the FU parental line carrying the allele of PR susceptibility and the positive allele for yield, collar diameter, number of leaves and M3 developmental stage. In the genetic background covered by the FU*PAZ2 population, a significant part of the genetic variation for susceptibility to PR was therefore found positively associated with vigor related traits. Although a few explanations could be proposed today for this result (see Additional file 4), it would be highly premature to put forward any hypothesis without further research. However, this result might open insights into the functional relationship between disease resistance and phenotype related traits.

## Conclusions

This work shows for the first time the quantitative behaviour of the sunflower resistance to Premature Ripening caused by *P. macdonaldii*. It also brings evidences that different genetic factors are implicated in the disease development, depending on the infection process leading to black stem or premature ripening diseases, and on the stage of disease development. Although phenotyping for PR resistance appears hard because it must be done on adult plants in field conditions according to an ordinal scale [34], QTL involved in resistance to premature ripening have been successfully detected.

A high quality reference sequence of *H. annuus* is now available [35], based on the parental line XRQ. Moreover, an AXIOM array including more than 586 K SNP [36] has been built from that sequence, and high density polymorphism data are now available for 72 sunflower lines, including the parental lines FU, XRQ, PAZ2 and PSC8. Therefore, there is now more possibilities, including positional cloning, to decipher the genetic components involved in {*H. annuus* * *P. macdonaldii*} interaction and to open new prospects in sunflower breeding to improve resistance.

## Additional files

Additional file 1: Analyses of variance of the *Phoma macdonaldii* black stem disease and premature ripening scores and AUDPC in the two sunflower RIL populations XRQxPSC8 and FUxPAZ2. (XLSX 17 kb)

Additional file 2: Context sequences for AXIOM markers. (XLSX 63 kb)

Additional file 3: Genetic maps built on XRQxPSC8 and FUxPAZ2 RIL populations from a framework consensus genetic map. (XLSX 122 kb)

Additional file 4: Genetic architecture of the relationship between morphological and developmental traits, and sunflower resistance traits to *Phoma macdonaldii.* (DOCX 28 kb)

## Abbreviations

AUDPC: Area under progress curve
BS: Black stem disease
DPI: Day post-inoculation
LG: Linkage group
PR: Premature ripening
QTL: Quantitative trait loci
RIL: Recombinant inbred line
