# Supplementary material for: Different genetic architectures underlie crop responses to the same pathogen: the {Helianthus annuus * Phoma macdonaldii} interaction case for black stem disease and premature ripening
Source: BMC Plant Biol. 2017 Oct 19;17:167. doi: 10.1186/s12870-017-1116-1 (PMC5649070; doi:10.1186/s12870-017-1116-1)
Supplement: Supplementary file 4 — Genetic architecture of the relationship between morphological and developmental traits, and sunflower resistance traits to Phoma macdonaldii. (DOCX 28 kb) [file 12870_2017_1116_MOESM4_ESM.docx]

**Additional File 4:** Genetic architecture of the relationship between morphological and developmental traits, and sunflower resistance traits to *Phoma macdonaldii.*

**INTRODUCTION**

Independently from the arsenal of genes directly involved in plant response to the pathogen, morphological and developmental traits may affect the disease development. The relationship between such traits and disease resistance traits may occur through environmental conditions, including crop management. For example, drought and high nitrogen input have been found favorable for the Premature Ripening disease caused by *P.macdonaldii* [1, 2]. The fraction of photosynthetically active radiation intercepted by the sunflower canopy has been shown a good indicator to predict the development of sunflower lesions caused by *Phomopsis helianthi* [3]. This relationship may also occur through genetic correlation. For example, it has been demonstrated [4], by QTL mapping in a RIL population, a tight genetic relationship between plant architecture and heading date, and resistance to sheath blight in wheat. As morphological and developmental traits were available for the FU*PAZ2 RIL population used in the main manuscript, we made in this supplementary study a comparison of the QTL map for morphological and developmental traits, and *P.macdonaldii* disease related traits.

**Materials and Methods**

***Sunflower genetic material and experimental design:***

A field experiment was carried out in 2011 without artificial inoculation to evaluate architectural, developmental traits and grain yield (*per se*, under open pollination) on 124 RIL from the FUxPAZ2 RIL population (See main manuscript for more information). An incomplete lattice design (12*12) with 3 replicates was used for this trial, including replicated checks across the design. Each plot consisted of 3 rows of about 25 plants. The experiment was planted on May 10. Plant density was 7 plants.m^-2^ after thinning. No natural infection by *P. macdonaldii* was observed in this trial. Degree days (basis 6°C) to F1, M0 and M3 developmental stages [5], plant height, collar diameter, total number of leaves, length, width and rank of the biggest leaf were individually recorded on 5 plants per plot. Each plot was harvested at maturity to record the grain yield.

***Statistical analyses and QTL mapping:***

Adjusted means for each trait were produced by GLM procedure (SAS software, SAS Institute Inc.). QTL mapping was done using the same procedure than for disease related traits (See main manuscript).

**RESULTS AND DISCUSSION**

***Colocalization of QTL involved in morphogical traits and disease related traits:***

Several QTL involved in architectural traits like the number of leaves, the stem base diameter and at some extent the plant height, are located in the same genetic map area (Table S3.1 and Figure 8 of the main manuscript), suggesting a strong pleiotropic effect. In the same genetic map area, a QTL accounting for 26.7% of the phenotypic variation for the grain yield (in open pollination, *per se* evaluation) was found co-localized with the QTL of PR resistance on LG7. The FU parental line was found carrying the allele of PR susceptibility and the positive allele for yield, collar diameter, number of leaves and M3 developmental stage. The positive correlation between PR susceptibility and grain yield could be also observed from raw data, as a statistically significant correlation was found between grain yield and AUDPC_PR of RIL (R2=0.22, p-value <0.01).

No colocalization with morphological traits was found for resistance to BS. This result is another indication showing that, in the FU*PAZ2 genetic background, not the same genetic factors are involved for resistance to PR and BS (see main manuscript).

A positive correlation has been found [2] between the plant stem diameter and the plant ability to resist to premature ripening. Actually, there is no contradiction between these results and ours, as [2] considered environmental variation (same genotype, different crop management) whereas we considered genetic variation (one crop management, genetic variation between RIL).

Different explanations could account for our results. Firstly, it might be hypothesized that the two alleles at this QTL are accounting for alternative ways in sugar partitioning towards growth or grain filling *vs*. reinforcement of structural tissues including physical barriers making them able or not to delay the invasion of the collar and root system by the fungus. Such a scenario has been demonstrated in *Populus tremuloides* [6], where under elevated CO2, two different clones were found, by transcriptomic analysis, allocating carbon either to starch biosynthesis and growth, or to passive defense through the phenylpropanoid pathway. Secondly, the genotypes having the FU allele at this QTL might mainly devote the available resources to the aerial growth and development, thus allowing a good seed set in well-watered and healthy environmental conditions, and not developing enough their root system to prevent the drought stress. Indeed, the drought stress has been found increasing the impact of the PR caused par *P. macdonaldii* since the fungus is able to colonize xylem vessels and increases the risk of vascular embolisms under drought conditions [1]. In order to test these hypotheses, it would be necessary to compare two sets of RIL carrying one or the other flanking marker through physiological and cytological approaches.

***Impact of developmental stages on the disease assessment:***

Two QTL accounting globally for 27% of the phenotypic variation were found for flowering time (F1, non-inoculated conditions) on LG11 and LG14 in the FUxPAZ2 RIL population. As no QTL was found on these linkage groups for PR or BS disease scores, it seems therefore likely that the differential response of RIL to PR or BS is not associated, in this population, to differences in earliness of flowering time. Moreover, the linkage groups carrying QTL involved in quantitative resistance (LG7 and LG13) were not found carrying QTL involved in the genetic architecture of earliness in a previous study [7]. A QTL for the M3 developmental stage was detected at the same position as the QTL for the late symptoms related traits of PR resistance. The allele of the line FU is conferring better yield (see above), latter physiological maturity, and higher susceptibility. This suggests at some extent that the experiment described in this Additional File, where no artificial infection was done, was effectively not contaminated by a natural infection. Indeed, otherwise, a natural infection would have resulted in a premature ripening leading to an earlier physiological maturity.

**ReferenceS**

1. Seassau C, Dechamp-Guillaume G, Mestries E, Debaeke P: **Nitrogen and water management can limit premature ripening of sunflower induced by *Phoma macdonaldii.*** *Field Crops Research* 2010, **115(1):**99-106.
2. Seassau C, Dechamp-Guillaume G, Mestries E, Debaeke P: **Low plant density can reduce sunflower premature ripening caused by *Phoma macdonaldii***. *European Journal of Agronomy* 2010, **43:**185-193.
3. Debaeke P, Estragnat A: Crop canopy indicators for the early prediction of Phomopsis stem canker (*Diaporthe helianthi*) in sunflower. Crop Protection 2009, 28 (9): 792-801.
4. Guo Y, Du ZY, Chen J, Zhang Z: **QTL mapping of wheat plant architectural characteristics and their genetic relationship with seven QTLs conferring resistance to sheath blight**. *Plos One* 2017, **12(4)**: e0174939.
5. Merrien A: *Physiologie du tournesol*. Paris (France): CETIOM; 1992.
6. Cseke LJ, Tsai CJ, Rogers A, Nelsen MP, White HL, Karnosky DF, Podila GK: **Transcriptomic comparison in the leaves of two aspen genotypes having similar carbon assimilation rates but different partitioning patterns under elevated [CO2]**. *New Phytologist* 2009, **182:**891-911.
7. Cadic E, Coque M, Vear F, Grezes-Besset B, Pauquet J, Piquemal J, Lippi Y, Blanchard P, Romestant M, Pouilly N, Rengel D, Gouzy J, Langlade N, Mangin B, Vincourt P: **Combined linkage and association mapping of flowering time in Sunflower (*Helianthus annuus* L.)**. *Theor Appl Genet* 2013, **126(5)**:1337-1356.

**Table S3.1.** QTL detected (Type I error rate of 5 % at the genomewide level) for morphological and developmental traits and yield *per se* in the FUxPAZ2 population. See also Figure 8 in the main manuscript.

| **Traits** | **LG** | **position** | **min** | **max** | **R2** | **MCQTL Test** | **LOD** | **FU-PAZ2  contrast: value** | **FU-PAZ2  contrast: unit** |
| --- | --- | --- | --- | --- | --- | --- | --- | --- | --- |
| Height | 7 | 23.7 | 0.0 | 53.9 | 14.5 | 4.9 | 4.50 | 6.2 | cm |
| Collar_diam | 7 | 31.4 | 21.9 | 49.5 | 23.8 | 8.0 | 8.21 | 1.2 | mm |
| Collar_diam | 10 | 109.9 | 96.6 | 113.3 | 16.4 | 5.5 | 5.15 | -1.00 | mm |
| Leaf_Rk | 11 | 65.1 | 40.7 | 103.9 | 13.7 | 4.6 | 4.18 | -1.60 | % |
| Leaf_Rk | 14 | 44.1 | 21.6 | 70.8 | 18.0 | 6.0 | 5.77 | -2.00 | % |
| Nb_Leaves | 7 | 38.9 | 15.8 | 54.1 | 14.2 | 4.7 | 4.32 | 1.20 | number |
| Nb_Leaves | 10 | 66.9 | 59.9 | 78.5 | 20.5 | 6.8 | 6.74 | -1.50 | number |
| Nb_Leaves | 14 | 72.9 | 63.1 | 80.8 | 29.2 | 9.9 | 10.75 | 2.10 | number |
| Leaf_Length | 7 | 49.9 | 44.3 | 54.0 | 17.9 | 6.0 | 5.73 | 1.00 | cm |
| Leaf_Length | 10 | 109.9 | 54.6 | 113.0 | 17.4 | 5.8 | 5.56 | -0.80 | cm |
| Leaf_Width | 7 | 49.9 | 25.2 | 54.4 | 20.7 | 7.0 | 6.94 | 1.20 | cm |
| F1 | 11 | 120.4 | 110.3 | 126.1 | 14.5 | 4.9 | 4.46 | 18.40 | degree * days |
| F1 | 14 | 72.9 | 59.1 | 80.8 | 18.0 | 6.0 | 5.76 | 19.50 | degree * days |
| M0 | 11 | 123.4 | 106.2 | 126.3 | 14.3 | 4.8 | 4.38 | 28.00 | degree * days |
| M0 | 14 | 80.8 | 61.8 | 80.8 | 12.7 | 4.3 | 3.83 | 20.00 | degree * days |
| M3 | 7 | 42.5 | 36.8 | 51.4 | 13.5 | 4.6 | 4.15 | 30.00 | degree * days |
| Yield | 7 | 42.5 | 38.6 | 44.8 | 26.7 | 9.1 | 9.69 | 4.00 | 0,1 t/ha |
